# T1 and extracellular volume mapping in the heart: estimation of error maps and the influence of noise on precision

**DOI:** 10.1186/1532-429X-15-56

**Published:** 2013-06-21

**Authors:** Peter Kellman, Andrew E Arai, Hui Xue

**Affiliations:** 1 National Heart, Lung, and Blood Institute, National Institutes of Health, DHHS, 10 Center Drive MSC-1061, Bethesda, MD 20892, USA

**Keywords:** T1 Map, Error, Precision, MOLLI, Extracellular, Diffuse Fibrosis, Cardiovascular Magnetic Resonance

## Abstract

**Background:**

Quantitative measurements in the myocardium may be used to detect both focal and diffuse disease processes that result in an elevation of T1 and/or extracellular volume (ECV) fraction. Detection of abnormal myocardial tissue by these methods is affected by both the accuracy and precision. The sensitivity for detecting abnormal elevation of T1 and ECV is limited by the precision of T1 estimates which is a function of the number and timing of measurements along the T1-inversion recovery curve, the signal-to-noise ratio (SNR), the tissue T1, and the method of fitting.

**Methods:**

The standard deviation (SD) of T1 and ECV estimates are formulated and SD maps are calculated on a pixel-wise basis using the Modified Look-Locker Inversion recovery (MOLLI) method. SD estimates are validated by numerical simulation using Monte-Carlo analysis and with phantoms using repeated trials. SD estimates are provided for pre- and post-contrast optimized protocols for a range of T1s and SNRs. In-vivo examples are provide for normal, myocarditis, and HCM in human subjects. The formulation of SD maps was extended to R1 and ECV.

**Results:**

The measured myocardial SNR ranged from 23 to 43 across the heart using the specific T1-mapping protocol in this study. In this range of SNRs, the estimated SD for T1 was approximately 20-45 ms for pre-contrast myocardial T1 around 1000 ms, and was approximately 10-20 ms for post contrast T1 around 400 ms. The proposed estimate of SD was an unbiased estimate of the standard deviation of T1 validated by numerical simulation and had > 99% correlation with phantom measurements. The measured SD maps exhibited variation across the heart due to drop off in surface coil sensitivity as expected for the variation in SNR. Focal elevation in T1 and ECV was shown to have statistical significance on a pixel-wise basis for in-vivo examples.

**Conclusions:**

Pixel-wise estimates of T1 mapping errors have been formulated and validated, and the formulation has been extended to ECV. The ability to quantify the measurement error has potential to determine the statistical significance of subtle abnormalities that arise due to diffuse disease processes involving fibrosis and/or edema and is useful both as a confidence metric for overall quality, and in optimization and comparison of imaging protocols.

## Background

Quantitative methods such as T1-mapping and extracellular volume (ECV) mapping appear promising to complement LGE imaging in cases of more homogeneously diffuse disease which affect the myocardial extracellular space [1-17]. Quantitative measurements in the myocardium may be used to detect both focal and diffuse disease processes that result in an elevation of T1 and/or ECV.

Direct measurement of extracellular volume (ECV) was initially developed for quantifying the myocardial extracellular fractional distribution volume [1] and has been proposed as a means for detection and quantification of diffuse myocardial fibrosis [2-6,15-17]. This approach is based on the change in T1 following administration of an extracellular contrast agent and circumvents the limitation of LGE in detecting a global change in T1, which typically uses a single post-contrast T1 measurement. The myocardial ECV is measured as the percent of tissue comprised of extracellular space, which is a physiologically intuitive unit of measurement. ECV has been shown to correlate with collagen volume fraction [3,5]. The topic of ECV mapping as well as native T1-mapping is of current interest as a diagnostic tool for a wide range of cardiomyopathies including dilated cardiomyopathy [2], diffuse fibrosis associated with myocardial dysfunction in congenital heart disease [4], aortic stenosis and HCM [5], cardiac amyloidosis [15], Anderson Fabry [17], myocardial infarction [8,11], as well as aging processes [8].

The sensitivity for detecting abnormal elevation of T1 and ECV is limited by the precision of T1 estimates which is a function of the number and timing of measurements along the T1-inversion recovery curve, the signal-to-noise ratio (SNR), the tissue T1, and the method of fitting. Detection of abnormal myocardial tissue by these methods is affected by both the accuracy and precision. In this work, we only consider the random component due to noise which limits precision and not bias errors that affects accuracy. Although absolute accuracy of in-vivo measurements is an important and open subject, these methods have been shown to be highly reproducible [18] in practice despite bias errors.

Quantifying the statistical fluctuation of measured T1 could improve confidence in assessing the significance of results. We propose to produce a map calibrated in T1 units that represents the quality of the T1 estimate, by transforming the standard deviation (SD) of residual fitting error into the SD of the estimated parameters. The estimate of the T1 parameter error based on the fit residuals is derived analytically, and validated on phantom measurements. Robust methods for both fitting and estimating the SD of the underlying measurements are described. In-vivo examples are shown to demonstrate the potential utility.

## Methods

### Theory

The sensitivity for detecting abnormal elevation of T1 is limited by the precision of T1 estimates, which in the case of inversion recovery methods is a function of the number and timing of measurements along the T1-recovery curve, the signal-to-noise ratio, tissue T1, method of fitting, and the accuracy of the model. Physiologic fluctuation is not considered. It is proposed to produce a standard deviation (SD) map calibrated in T1 units that represented the standard deviation of the T1 estimate, by transforming the SD of the residual fitting error into the SD of the estimated parameters. Estimate of the T1 parameter error based on fit residuals is derived analytically, and validated by both Monte-Carlo numerical simulation and phantom measurements using repeated trials.

**Figure 1:**
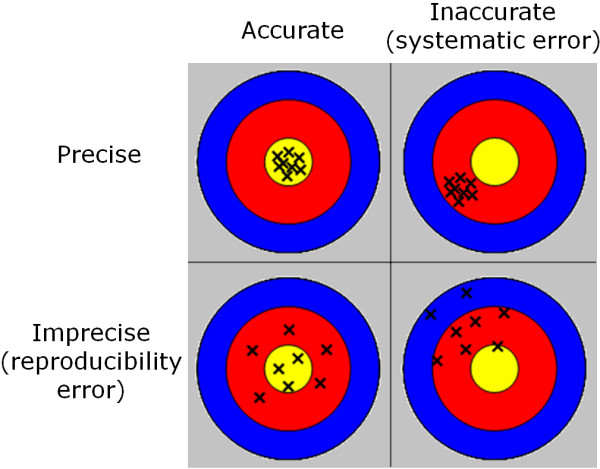
Illustration of accuracy versus precision.

Inversion recovery is widely used for T1-mapping using Look-Locker methods. In applications such as cardiac MR, a modified Look-Locker (MOLLI) method [9,10] uses inversion recovery with multiple single shot images at different inversion times. Pixel-wise parametric mapping is accomplished by performing a curve fit to the multiple inversion time measurements. The original MOLLI paper [9] assumes a 3-parameter model of the form S(ti) = A – B exp(-ti/T1*), where T1* < T1 represents the apparent T1 which is shortened by the influence of imaging RF pulses. The desired T1 is then calculated at each pixel using T1 = T1*·(B/A-1), referred to as the Look-Locker correction, originally derived from considering a continuous fast low angle shot (FLASH) gradient echo readout [19]. The Look-Locker correction is used in MOLLI despite the fact that imaging uses non-continuous balanced steady-state free precession (bSSFP), which violates the assumption used in the formulation. This assumption is a key source of bias error not treated in this paper and is sensitive to variables such as T1 and T2 as described in the literature [9,20,21]. In this work, we only consider the random component due to noise which limits precision and not bias errors that affect accuracy (Figure 1).

A phase sensitive (PSIR) reconstruction was used [22] to restore the sign and thereby avoid performing a magnitude fit, or performing multiple fits to estimate the zero crossing [9]. In PSIR reconstruction, the real component is used which results in normally distributed noise [23]. The 3-parameter model may be written as:

(1) $$y\left(t\right)=A-B\;\cdot \;\exp\left(-\frac{t}{T1^{*}}\right)=A-B\;\cdot \;\exp\left(-\frac{t\left(B/A-1\right)}{T1}\right)$$

and the measurements y(t) may be fit for the unknown parameters (A, B, T1) at each pixel using a downhill simplex minimization approach (Nelder–Mead method) [24].

The desired covariance matrix C of the estimated parameters (A, B, T1) may be approximated as [25]:

(2) $$C=\mathrm{inv}\left(D_{1/2}\right)=\left[\begin{array}{ccc} \sigma_{T1}^{2} & \cdot & \cdot \\ \cdot & \sigma_{A}^{2} & \cdot \\ \cdot & \cdot & \sigma_{B}^{2} \end{array}\right]$$

where the matrix **D**_1/2_ is a first order approximation to the Hessian matrix **D**. **D** is comprised of second order derivates of the chi-square cost to the parameters. **D**_1/2_ is computed as:

(3) $$D_{1/2}=\sum_{i=0}^{N-1}\frac{1}{\sigma_{i}^{2}}\left[\begin{array}{ccc} \frac{\partial y\left(t_{i}\right)}{\partial T1}\cdot \frac{\partial y\left(t_{i}\right)}{\partial T1} & \frac{\partial y\left(t_{i}\right)}{\partial T1}\cdot \frac{\partial y\left(t_{i}\right)}{\partial A} & \frac{\partial y\left(t_{i}\right)}{\partial T1}\cdot \frac{\partial y\left(t_{i}\right)}{\partial B}\\ \frac{\partial y\left(t_{i}\right)}{\partial A}\cdot \frac{\partial y\left(t_{i}\right)}{\partial T1} & \frac{\partial y\left(t_{i}\right)}{\partial A}\cdot \frac{\partial y\left(t_{i}\right)}{\partial A} & \frac{\partial y\left(t_{i}\right)}{\partial A}\cdot \frac{\partial y\left(t_{i}\right)}{\partial B}\\ \frac{\partial y\left(t_{i}\right)}{\partial B}\cdot \frac{\partial y\left(t_{i}\right)}{\partial T1} & \frac{\partial y\left(t_{i}\right)}{\partial B}\cdot \frac{\partial y\left(t_{i}\right)}{\partial A} & \frac{\partial y\left(t_{i}\right)}{\partial B}\cdot \frac{\partial y\left(t_{i}\right)}{\partial B} \end{array}\right]$$

after having dropped the second order terms in **D**. **D**_1/2_ is comprised of the partial derivatives of the signal Eq (1) relative to the estimated parameters, derived analytically for each inversion time t_i_ as:

$$\frac{\partial y}{\partial A}=1-B\;\cdot \;\exp\left(-\frac{t\left(B/A-1\right)}{T1}\right)\cdot \frac{t\cdot B}{T1\cdot A^{2}}$$

(4) $$\begin{array}{l} \frac{\partial y}{\partial B}=-\exp\left(-\frac{t\left(B/A-1\right)}{T1}\right)\\ \;+B\cdot \exp\left(-\frac{t\left(B/A-1\right)}{T1}\right)\cdot \frac{t}{T1\cdot A} \end{array}$$

$$\frac{\partial y}{\partial T1}=-B\;\cdot \;\exp\left(-\frac{t\left(B/A-1\right)}{T1}\right)\cdot \frac{t\cdot \left(B/A-1\right)}{T1^{2}}$$

The noise of the PSIR reconstructed measurements is normally distributed with standard deviation σ_i_ for each measurement which can be treated as equal across measurements (σ_i_ = σ) and are estimated at each pixel from the fit residuals. Although the measurements are very well approximated as normal, the parameter estimates themselves may not be normally distributed due to the non-linearity of the model. However, in our experience the parameter estimates (A, B, and T1) were well approximated as normal in the range of SNR encountered.

The above formulation may also be extended to ECV maps, where ECV is calculated as:

(5) $$\mathrm{ECV}=\left(1-\mathrm{HCT}\right)\frac{{\left(R1_{\mathrm{post}}-R1_{\mathrm{pre}}\right)}_{\mathrm{myocardium}}}{{\left(R1_{\mathrm{post}}-R1_{\mathrm{pre}}\right)}_{\mathrm{blood}}}$$

In this case, it is assumed that the random noise component in the ECV measurement is dominated by the myocardium since the R1 in the blood is measured in a large ROI, which reduces the noise. The calculation of SD is reformulated for R1 = 1/T1 by substituting δy/δR1 for δy/δT1 in Eq (3) above.

(6) $$\frac{\partial y}{\partial R1}=t\cdot \left(B/A-1\right)\cdot B\;\cdot \;\exp\left(-t\cdot \left(B/A-1\right)\cdot R1\right)$$

The SD for ECV is calculated as:

(7) $$\sigma_{\mathrm{ECV}}=\frac{\left(1-\mathrm{HCT}\right)}{\Delta R1_{\mathrm{blood}}}\cdot \sqrt{\sigma_{R1\mathrm{post}}^{2}+\sigma_{R1\mathrm{pre}}^{2}}$$

which is dominated by the post-contrast variation, i.e. σ_R1post_., due to the increased value of R1 following administration of contrast.

### Robust estimation

Robust estimation was used for both fitting for T1 as well as for estimation of the standard deviation, σ, of the measurements. Robust fitting [26,27] uses iterative re-weighting to improve the fit in the presence of outliers. At each iteration, the weighting of outliers is reduced based on the value of their residuals. For instance, outliers may result from errors such as residual uncorrected motion. Additionally, a robust estimation of the underlying standard deviation (σ) from the fit residuals is used based on the median absolute deviation (MAD) approach [26,28]. The MAD estimate σ is calculated from the residuals ϵ_i_ = (fit – meas) as:

(8) $$\sigma =\mathrm{median}\left(\mathrm{abs}\left(r_{i}\right)\right)/0.6745$$

where r_i_ are the residuals ϵ_i_ after discarding the (p-1) values with lowest magnitude, p = 3 is the number of parameters being fit, and the scale factor 0.6745 is used for noise which is normally distributed. The scale factor is calculated as the median of the absolute value of normally distributed noise with standard deviation equal to 1. Discarding the lowest residuals is necessary to avoid a bias error due to over fitting.

**Figure 2:**
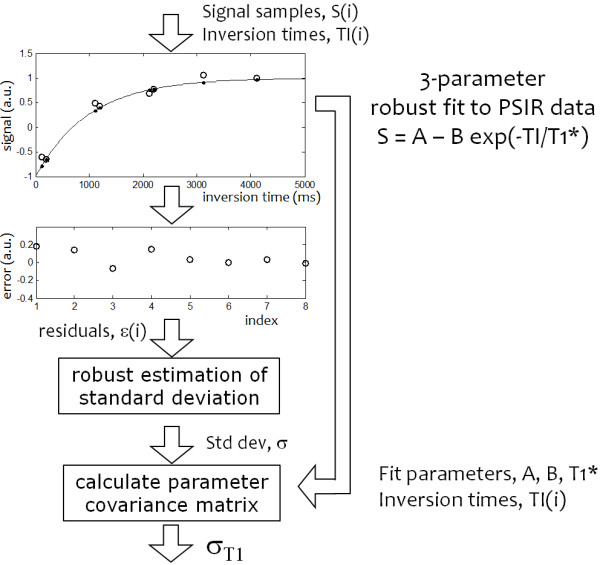
Pixel-wise calculation of SD values based on fit residuals.

The estimated SD for the corresponding T1-map value is calculated at each pixel producing a SD map, following the flow chart in Figure 2.

### Numerical validation

A Monte-Carlo simulation using N = 65,536 trials was used to compute the standard deviation in T1 (σ_T1_) as a function of SNR, and T1 for a specific MOLLI protocol (5-3 sampling, TImin = 105 ms, TI shift = 80 ms) and T2 = 45 ms, and was compared with the estimate of standard deviation based on the proposed approach using the fit residuals. Each trial used independent noise with a normal distribution. In order to test the robustness to outliers, a separate test was conducted which compared the robust iteratively re-weighted and standard methods in the presence of outliers. For this comparison, a single outlier with a randomly positioned inversion time (i.e., 1 of the 8 measurements) had a noise standard deviation corresponding to 6 times the noise standard deviation of the other measurements.

In addition to measuring the accuracy of the estimate of SD, i.e. whether the estimate is unbiased, the variability or standard deviation of the standard deviation estimate was measured.

### Phantom validation

Experimental validation was performed using phantoms by repeated measurements (n = 200 repetitions) of a set of T1-measurements comparing estimated and calculated standard deviations. A “measured” SD was calculated for each pixel, and compared with the mean of the estimated SD map values for the corresponding pixel. Phantom validation used a set of CuSO4 doped agar gel phantoms with varying concentrations with T1 and T2 in the expected range for myocardium, both native and with Gd contrast. Phantoms had T1 in the range 250-1600 ms and T2 in the range 40-75 ms.

### Imaging

Imaging was performed on 32 channel 1.5 T Siemens Avanto and Espree scanners (Siemens Medical Solutions, Erlangen, Germany), equipped with 45 mT/m and 200 T/m/s, and 33 mT/m and 170 T/m/s gradient systems, respectively. The MOLLI imaging protocol used in this study acquired data with 2 protocols (sampling strategies). The first protocol was optimized for native (pre-contrast) myocardial T1 values and the second protocol was optimized for shorter T1 corresponding to Gd contrast. The MOLLI imaging protocol used for native T1 acquired data at 8 inversion times over an 11 heart beat breath-hold at end-expiration with 2 inversions. For the sake of reduced heart rate variability the protocol used a 5(3)3 scheme, acquiring 5 images after the first inversion, a 3 second pause for recovery, and 3 images acquired after the second inversion. The protocol used with Gd contrast had a 4(1)3(1)2 sampling scheme which acquired 9 images in 11 heartbeats with 3 inversions which was found to improve the T1 estimate. For the contrast protocol with shorter T1, a shorter recovery period (1s) is used between inversions, which permits additional samples of the inversion recovery at short inversion times in the same overall breath-hold duration.

Typical imaging parameters for both sampling schemes were: non-selective adiabatic inversion pulse, steady state free precession single shot read out with 35° excitation flip angle, typical field of view 360 × 270 mm^2^, slice thickness 6 mm, minimum inversion time 110 ms, inversion time increment 80 ms, 7/8 partial Fourier plus parallel imaging factor 2. A 2.56 ms tan/tanh adiabatic inversion pulse was used to improve the inversion efficiency at the low values of myocardial T2. For heart rates below 90 bpm we typically used: matrix 256 × 144, voxel size 1.4 × 1.9 × 6.0 mm^3^, TR/TE 2.7/1.1 ms, with 200 ms readout imaging duration. For heart rates above 90 bpm a matrix of 192 × 130 was used.

Images were reconstructed using a phase sensitive method which incorporates non-rigid correction of respiratory motion [22]. Co-registration between pre- and post-contrast acquired image series was performed in the generation of ECV maps [13]. T1 and associated SD-maps were generated using the PSIR MOCO method [22] and were processed in-line on the scanner, while R1, ECV, and SD-maps of ECV with co-registration were processed off-line.

### In-vivo studies

This study was approved by the local Institutional Review Boards of the National Heart, Lung, and Blood Institute and Suburban Hospital, and all subjects gave written informed consent to participate. Hematocrit used in calculation of ECV was measured from a venous blood sample drawn just prior to the CMR study. T1-maps were typically acquired at least 15 min following administration of Gd contrast (0.15 mmol/kg) (Gadavist, Bayer Healthcare). In-vivo data was acquired to (1) measure typical myocardial SNR, (2) to demonstrate the value of SD maps in assessing the statistical significance of subtle changes in T1, and (3) to illustrate the application to measurement of ECV mapping.

### SNR measurements

The SNR in the myocardium was measured in-vivo in n = 20 subjects prior to contrast in order to derive typical values of SNR used in the analysis. Images were reconstructed with scale in SNR units [29] and values were measured in manually traced septal and lateral wall ROIs. Reconstruction in SNR units [29] facilitates SNR measurement but requires off-line reconstruction from the raw acquired data. SNR maps were calculated from the signal intensities of the longest inversion time image which has achieved steady state. The MOLLI imaging protocol used for SNR measurement was modified to acquire 12 heartbeats to ensure complete recovery was achieved. The sampling for this protocol was 12(1)1, i.e, images acquired for 12 heartbeats on the first inversion, followed by 1 recovery beat, and acquisition of 1 additional heartbeat on the second inversion.

## Results

### SNR measurements

Myocardial SNR with the MOLLI protocol was measured in 20 subjects using a voxel size of 1.4 × 1.9 × 6 mm^3^. The SNR in the septum was 43 ± 11 (m ± SD) and in the lateral wall was 22.8 ± 4.3. The SNR of the LV blood pool averaged over the full cavity was 42.7 ± 7.9.

### Monte Carlo simulation

**Figure 3:**
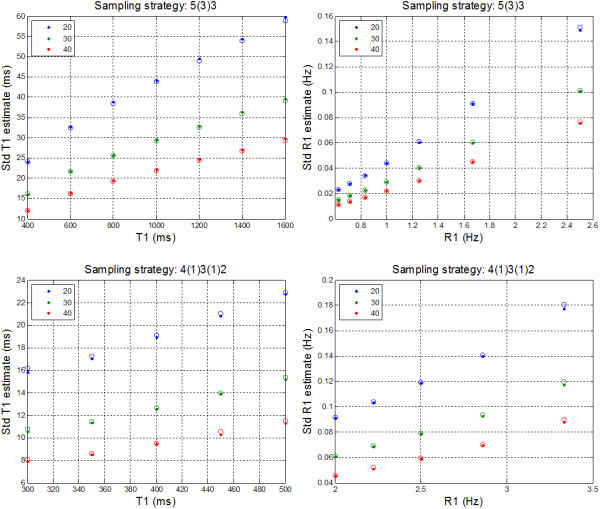
Measured (dots) vs calculated (circles) standard deviation of T1 vs T1 (left column) for various image SNRs for 2 sampling strategies, 5(3)3 and 4(1)3(1)2, from Monte-Carlo simulation with 65,536 trials, and corresponding standard deviations for R1 = 1/T1 (right column).

The measured and calculated estimates for T1 standard deviation as well as R1 = 1/T1 are graphed (Figure 3) for a range of T1 and SNR = 20, 30, and 40 which were in the range of SNRs measured using the specified imaging protocol. The 5(3)3 sampling strategy is simulated for T1 in the range 400-1600 ms, and the 4(1)3(1)2 sampling strategy is simulated for T1 in the range 300-500 ms. The estimated SD is within 1 ms of the calculated SD in all cases, i.e., the estimate is unbiased. The variability or standard deviation of the SD estimate was found to be related to the number of inversion time measurements used in the fit. For the 5(3)3 protocol with 8 measurements the standard deviation of the SD estimate was 1/3 of the mean SD value (mean over ROI), whereas for the 4(1)3(1)2 protocol with 9 measurements, the standard deviation of the SD estimate was 1/3.3 of the mean SD value. This variability in the distribution of errors was constant over the range of T1’s and SNR’s simulated. This variability corresponds to a single pixel and is reduced by sqrt(N) when averaging over a ROI with N independent samples which was verified in the Monte-Carlo simulation by averaging over blocks of variable length N.

**Figure 4:**
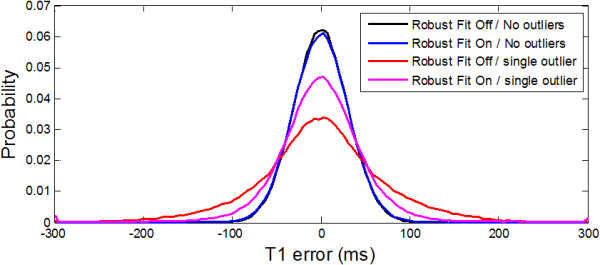
Histogram of T1 errors for case of T1 = 1000 ms, SNR = 30 illustrating the difference between iteratively re-weighted robust fitting in the presence of outliers.

To illustrate the robustness to outliers (Figure 4), the histogram of T1 measurements for a single protocol (T1 = 1100 ms, SNR = 20, 5(3)3 sampling) was performed with a single randomly located outlier at 6 standard deviations (n = 262,144 trials). Without outliers both the non-robust (plotted black) and robust (plotted blue) fitting have similar performance (SD = 30.3 and 31.3 ms, respectively). With a single random outlier the SD becomes 71.6 ms (plotted red) vs 56 ms for the iteratively re-weighted robust fitting (plotted magenta).

### Phantom measurements

**Figure 5:**
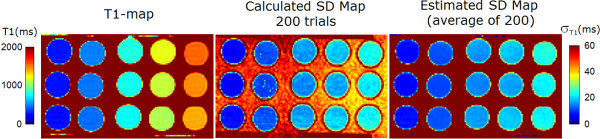
T1-map (LEFT) for CuSO4 doped agar gel phantoms for varying T1 and T2, corresponding SD map calculated from 200 trials (CENTER), and mean of estimated SD maps for 200 trials using proposed method (RIGHT).

**Figure 6:**
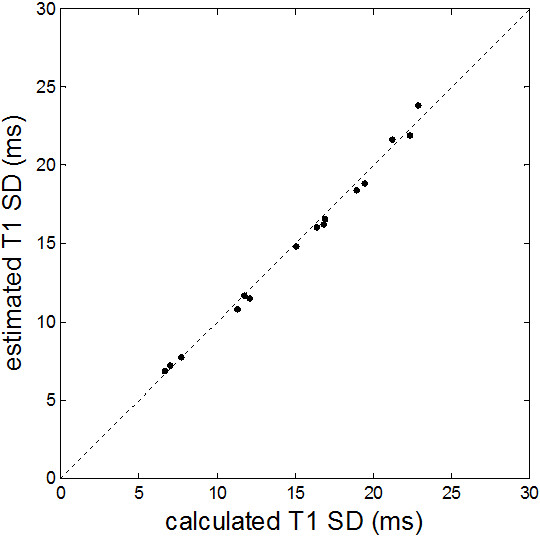
**Comparison of estimated (proposed method) versus calculated (200 repeated trials) SD for phantom data shown in Figure**5**.**

The proposed method of estimating SD was validated in agar gel phantoms using the method of repeated trials (Figure 5). The estimated values for SD correlated extremely well (r = 0.997) with the calculated values across a range of SD’s for tubes with varying T1, T2 and SNR (Figure 6).

### In-vivo examples

**Figure 7:**
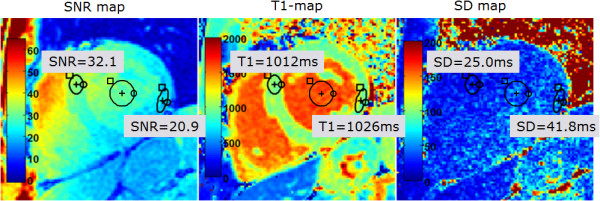
**SNR map (left), T1-map (center), and SD map (right).** Note the increased T1 standard deviation of T1 at the lateral wall corresponds to decreased SNR resulting from drop off of surface coil sensitivity.

An example T1-map for a normal subject (Figure 7) illustrates the variation in T1 estimation error with surface coil intensity variation. The anteroseptal ROI has SNR = 32.1, T1 = 1012 ms, SD = 25.0 ms, whereas the lateral wall ROI has lower SNR = 20.9, T1 = 1026 ms, SD = 41.8 ms (increased). The SNR in the LV blood pool ROI had T1 = 1580 ms, SNR = 29, and SD = 38 ms.

**Figure 8:**
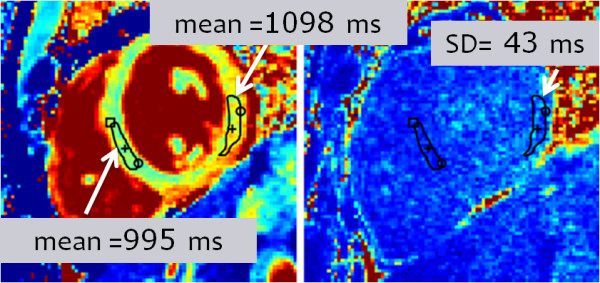
Example T1-map and SD map for subject with myocarditis and focal abnormality corresponding to T1 elevation (103 ms) between lateral wall and septum of 2.4 SD on a pixel-wise basis (lateral wall SD = 43 ms).

**Figure 9:**
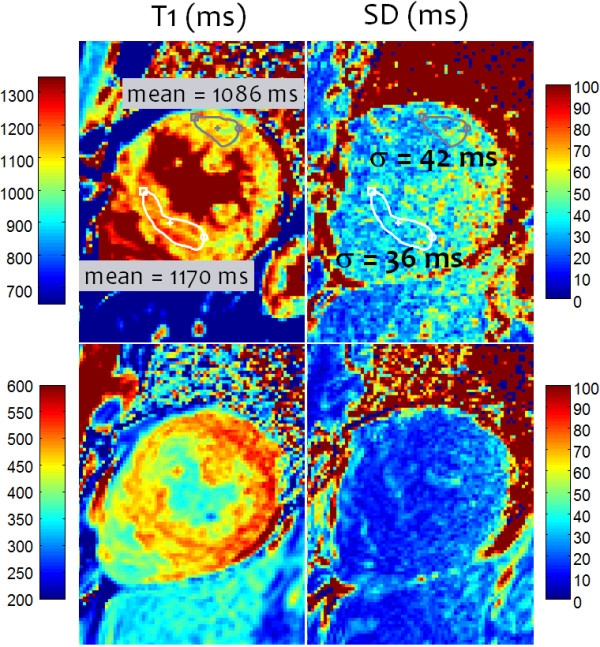
**Example T1 and SD maps for subject with HCM acquired pre-contrast (TOP) and post-contrast (BOTTOM).** Pre-contrast T1 maps exhibit focal T1 abnormalities in the septal region corresponding to T1 elevation of 84 ms relative to the lateral wall representing an elevation of 2.3 SD on a pixel-wise basis (septal SD = 36 ms).

Example T1-maps illustrate detection of subtle, focal elevation in T1 in subjects with myocarditis (Figure 8) and HCM (Figure 9). The subject with myocarditis (Figure 8) has a focal elevation of the sub-epicardial region of the lateral wall (1098 ms) corresponding to 103 ms elevation with respect to the septum (995 ms). The SD of in the lateral wall region is estimated to be 43 ms, i.e., the focal elevation is approximately 2.4 SD on a pixel-wise basis indicating statistical significance (P < 0.01) for this individual which has even greater significance on a ROI basis (see Discussion).

**Figure 10:**
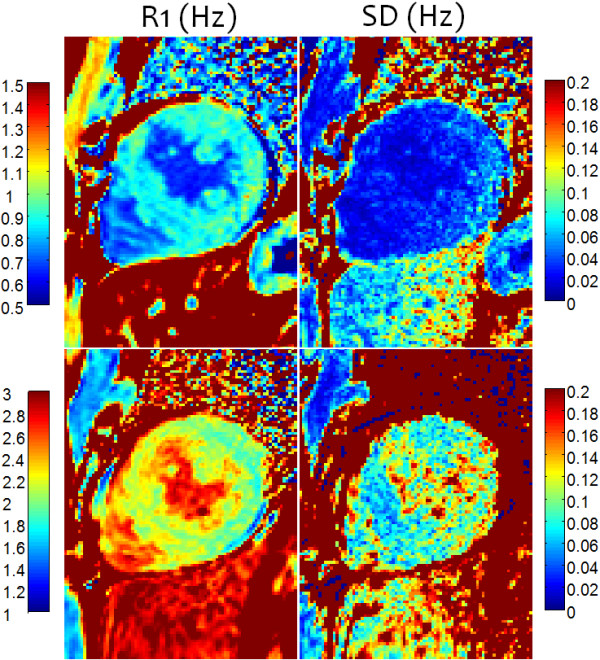
**R1 and SD maps for subject with HCM acquired pre-contrast (TOP) and post-contrast (BOTTOM), corresponding to the T1-maps shown in Figure**9**.**

**Figure 11:**
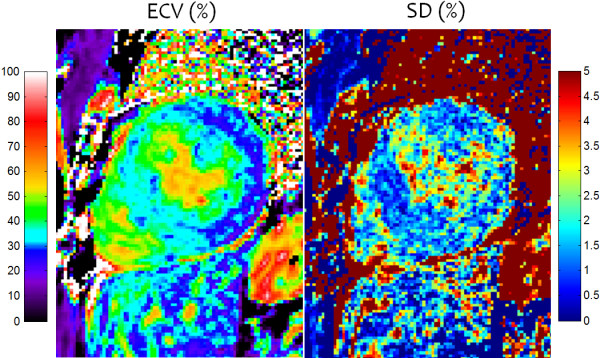
**ECV and SD maps for subject with HCM acquired corresponding to the R1-maps shown in Figure**10**, calibrated using LV blood and HCT values.**

The subject with HCM (Figure 9) has a focal elevation of the septal region (1170 ms) corresponding to 84 ms elevation with respect to the septum (1086 ms). The SD in the septal region is estimated to be 36 ms, i.e., the focal elevation is approximately 2.33 SD on a pixel-wise basis indicating statistical significance (P ≈ 0.01) assuming normal errors. The extension of error maps from T1 to R1 (Figure 10) and ECV (Figure 11) is illustrated for this subject with HCM. ECV and SD maps (Figure 11) are calculated from the ΔR1 maps after co-registration of pre- and post-contrast acquired maps [13] and scaling for HCT and ΔR1 of the blood pool. The ECV value in a septal ROI was 34.75% with SD estimated to be 1.0% and the lateral wall had ECV = 26.2 with SD = 1.2%. These SD values represent the pixel-wise variation.

**Figure 12:**
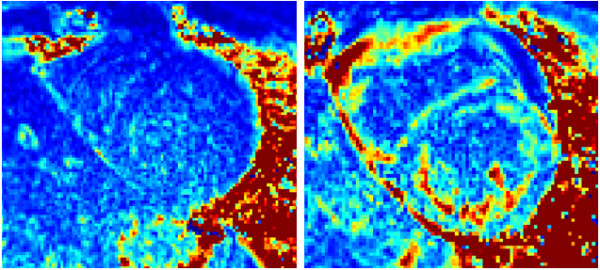
**Example SD maps for illustrating cases with increased error due to uncorrected motion of varying severity.** Uncorrected motion tends to cause local errors in the SD map which are readily recognized due to appearance of anatomical structure. The SD maps serve as an indication of T1-map quality.

Non-rigid motion correction used in these studies is designed to correct respiratory motion and is not as effective in correcting cardiac motion due to significant variation in the RR interval. In these cases, motion related changes in the signal will lead to increased error particularly around structures. Uncompensated motion related errors are readily apparent in the SD map (Figure 12) and may be used to assess the quality of the T1-map. For the case with more severe cardiac motion (Figure 12 right) the subject had a regular pattern of premature ventricular contractions (PVCs).

### Protocol Comparisons

**Figure 13:**
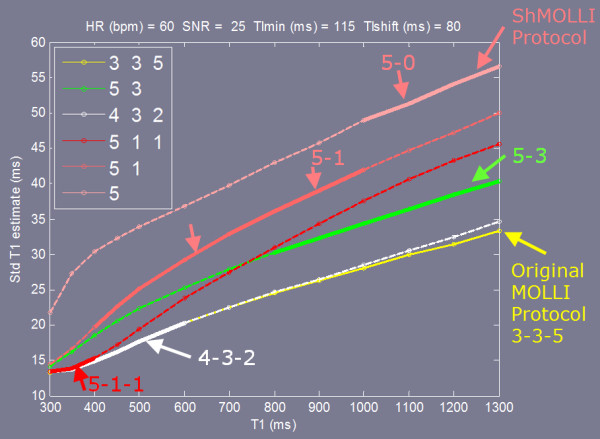
Comparison of the precision of various sampling strategies employed in widely used T1-mapping protocols.

The T1-mapping protocols 5(3)3 and 4(1)3(1)2 used in this study are compared in Figure 13 with the widely used original MOLLI protocol 3(3)3(3)5 and the shortened MOLLI protocol 5(1)1(1)1 [12] over a range of T1 at an SNR = 25. For the range of T1 values between 300 and 600 ms corresponding to subjects following administration of contrast, the 3(3)3(3)5, 4(1)3(1)2, and 5(1)1(1) are reasonably similar in performance. For longer values of T1 where it has been shown that the original MOLLI becomes heart rate dependent [11] the 5(3)3 and shortened MOLLI have been proposed to mitigate this. The shortened MOLLI reduces the dependence on heart rate by discarding samples depending on the measured RR interval. For the case of HR = 60 bpm shown in Figure 13, the precision of the Sh-MOLLI method is equivalent to the 5-0 sampling strategy for T1 > 1000 ms, and is equivalent to 5-1 between 400 and 1000 ms.

## Discussion

### Usefulness of the standard deviation measure

The clinician is frequently faced with the question of whether a regional variation or a global shift is real or is a random fluctuation due to noise. The proposed generation of an error map in meaningful units serves as an additional metric to aid in measuring statistical significance, as shown in the examples of Figures 8 and 9. As always, it is important to use judgement since there are other important sources of bias errors and artifacts. For example, regional variation in off-resonance can cause variation in apparent T1 which are treated here as bias errors since they do not increase the random fluctuation. This issue is important and must be considered in the assessment. The random component limiting precision is only a single component to consider, but serves as a performance bound. The proposed formulation may be used for predicting the precision of T1 and ECV estimates, and permits optimization and comparison of protocols.

#### Accuracy vs Precision

The issue of accuracy versus precision is an important subject. Although precision is essential to detecting subtle variations, the ability to detect global shifts in T1 is also limited by biases unless the normal reference values are reproducible, known and established. However, one must exercise caution since there are variable factors that may influence these “biases” such as off-resonance and shim, or protocol parameters such as matrix size. To some extent, the ECV may be less sensitive to accuracy in cases where the percentage error is the same for both myocardium and blood for both pre- and post-contrast acquisitions; in this case errors could cancel. In order to realize the full potential of T1 and ECV as biomarkers, bias errors should be minimized and the magnitude of any bias variation should be known.

#### SD estimation

Estimates of SD were found to be unbiased when using the MAD approach for estimating the standard deviation of fit residuals. The use of iteratively re-weighted fitting to be robust in the presence of outliers comes at a small penalty of a few percent in statistical efficiency in the case for which the data is normally distributed (see difference between blue and black plots in Figure 4 corresponding to 3% difference in SD). This sacrifice is deemed to be a small premium to pay for the gain in protection against outliers.

The largest source of errors in pixel wise T1-mapping is due to uncompensated heart motion due to variation in the RR interval, and this may be evident in the SD map. This increase in estimated SD due to motion error is apparent from structured appearance of the noise (Figure 12) and in this instance one must exercise caution in interpreting the T1-map values in these pixel regions which may be in error.

Although the formulation has been developed and validated for PSIR reconstructed images, this formulation may be extended to saturation recovery (SR) methods in cases where the SNR is reasonably high such that the noise distribution is sufficiently normal. It is not readily translated to magnitude IR T1-mapping due to the approximately Rician noise distribution in magnitude signal, particularly near the signal nulls. Furthermore, a frequently used implementation of the MOLLI method originally described [9,10] that uses the magnitude reconstructed images is based on a multi-fitting approach that perform multiple fits based on successfully incrementing the unknown time of zero-crossing and choosing the value with the best fit. This procedure is essentially estimating a 4-th parameter (the zero-crossing) which degrades the precision for specific values of T1 which have zero-crossings near the inversion times sampled [22].

The error in measuring T1 was found to be well approximated as a normal distribution, and therefore the analytic calculation agreed well with the Monte-Carlo results.

#### Pixel-wise T1 and SD mapping

T1-measurement on a pixel-wise basis provides the ability to detect regional heterogeneity and small focal elevations that might be difficult to identify using ROI based fitting. It also provides a context for identifying boundaries between blood and myocardium to mitigate partial volume contamination of ROI regions. Although from a noise standpoint the precision of fitting to ROIs is better than the pixel wise fit, the precision of the pixel-wise fits averaged over an ROI are similarly improved. In the in-vivo examples shown in Figures 8 and 9, the ROI sizes in the region of elevated T1 were 48 and 150 pixels, respectively. The SD of T1 on a ROI basis is improved by the square root of the number of independent pixels averaged. The number of independent pixels used to calculate the improvement in SD due to the averaging is somewhat less due to partial Fourier and parallel imaging factors and is estimated to be 40%, corresponding to 19 and 60 pixel averages, respectively. Thus the pixel-wise SD in Figure 8 example of 43 ms is approximately 10 ms on a ROI basis, further improving statistical significance.

SD maps for T1 and ECV may be used to provide confidence for gauging the statistical significance of abnormally elevated T1 and ECV on a pixel-wise basis, as well as indicating overall quality of the T1-map. Although the SD maps for current protocols are noisy in appearance, measurement of the SD in typical sized ROIs are dramatically improved in addition to the improvement in the actual T1 values. The ROI size for the septal ROI in the Figure 9 example was 150 pixels. Assuming that the actual number of independent samples was approximately 60 due to interpolated reconstruction, the SD estimate of 36 ms had a variability of 36/3/sqrt(60) = 1.5 ms.

## Conclusions

Pixel-wise estimates of T1 mapping errors have been formulated and validated, and the formulation has been extended to ECV. The ability to quantify the measurement error has potential to determine the statistical significance of subtle abnormalities that arise due to diffuse disease processes involving fibrosis and/or edema and is useful both as a confidence metric for overall quality, and in optimization and comparison of imaging protocols. The formulation provided may be extended to other parametric mapping measurements such as T2 or T2*.

## Abbreviations

CMR: Cardiovascular magnetic resonance; ECV: Extracellular volume fraction; LGE: Late gadolinium enhancement; MAD: Median absolute deviation; MOCO: Motion correction; MOLLI: Modified look-locker inversion recovery; TI: Inversion time; ROI: Region-of-interest; PSIR: Phase sensitive inversion recovery; SD: Standard deviation.

## Competing interests

Dr. Arai is a principal investigator on a US government Cooperative Research And Development Agreement (CRADA) with Siemens Medical Solutions (HL-CR-05-004).

## Authors’ contributions

PK conceived of the study, contributed to the formulation and evaluation of algorithms, performed processing and analysis, and drafted the manuscript. HX contributed to the formulation and evaluation of algorithms, and developed image reconstruction and mapping software. AEA participated in experimental design, was responsible for all human studies, and research funding. All authors participated in revising the manuscript and read and approved the final manuscript.
